# RNA contact prediction by data efficient deep learning

**DOI:** 10.1038/s42003-023-05244-9

**Published:** 2023-09-06

**Authors:** Oskar Taubert, Fabrice von der Lehr, Alina Bazarova, Christian Faber, Philipp Knechtges, Marie Weiel, Charlotte Debus, Daniel Coquelin, Achim Basermann, Achim Streit, Stefan Kesselheim, Markus Götz, Alexander Schug

**Affiliations:** 1 Steinbuch Centre for Computing (SCC), Karlsruhe Institute of Technology, 76344 Eggenstein-Leopoldshafen, Germany; 2 Institute for Software Technology (SC), German Aerospace Centre (DLR), 51147 Köln, Germany; 3 Jülich Supercomputing Centre, Forschungszentrum Jülich, 52428 Jülich, Germany; 4 Helmholtz AI, 81675 Munich, Germany; 5 Faculty of Biology, University of Duisburg-Essen, 45117 Essen, Germany

**Keywords:** Machine learning, Molecular modelling

## Abstract

On the path to full understanding of the structure-function relationship or even design of RNA, structure prediction would offer an intriguing complement to experimental efforts. Any deep learning on RNA structure, however, is hampered by the sparsity of labeled training data. Utilizing the limited data available, we here focus on predicting spatial adjacencies ("contact maps”) as a proxy for 3D structure. Our model, BARNACLE, combines the utilization of unlabeled data through self-supervised pre-training and efficient use of the sparse labeled data through an XGBoost classifier. BARNACLE shows a considerable improvement over both the established classical baseline and a deep neural network. In order to demonstrate that our approach can be applied to tasks with similar data constraints, we show that our findings generalize to the related setting of accessible surface area prediction.

## Abstract (web summary)

BARNACLE improves RNA contact prediction with limited labeled data, and such contacts can be used as restraints in RNA structure prediction.

## Introduction

Utilizing the limited available high-quality annotated data efficiently enables training deep-learning models in a range of scientific domains. In the molecular life sciences, the potential of complex models driven by big data has been demonstrated recently in protein structure prediction. In this work, we want to address the specific example of RNA contact prediction, for which the available labeled data is orders of magnitude smaller than for proteins. Accordingly, while there is currently significant work and progress on protein structure prediction, RNA has been and is receiving far less attention, in spite of the structure and function of functional RNA as part of the genome’s “dark matter” being just as vital and far less understood. While explored here in the context of RNA, our approach of applying a general machine learning technique to the fine-tuned latent representation of a pre-trained model can be generalized to not just other problems in molecular biology but any field. Let us first have a more detailed look into the biological context.

On the atomic level, life is realized via interacting biomolecules. To understand a biomolecule’s function in detail, one must know its specific biomolecular structure. Structure determination techniques have enabled an increasingly detailed understanding of nanoscale processes, yet many important biomolecules have not yet been structurally resolved. In the last decade, protein structure prediction techniques have increasingly complemented experimental efforts^1–9^, culminating in the development of powerful deep-learning techniques such as DeepMind’s AlphaFold 2 system^8^ (AF2) which is already driving many applications^10–12^. These techniques profit from the wealth of well-annotated protein databases on both the structure and sequence levels. Yet, only 1–2% of the human genome encodes proteins and we know little about the remaining 98%, the genome’s “dark matter” which encodes, e.g., structural Ribonucleic acids (RNA) and performs many critical regulatory functions^13^.

RNA are a crucial class of biomolecules which—similar to proteins—play essential roles in many fundamental processes. While most known as passive carrier of genetic information, RNA has been attributed many additional biological functions. The diversity of RNA function is so complex that it has been argued that life itself started with RNA^14^. However, many noncoding RNA still have unknown functions^15^ even today. Currently, dedicated focus has been placed on RNA for technological application^16^ and medical interest^17^, especially for the development of vaccines in light of the ongoing COVID-19 pandemic^18,19^.

RNA are composed of linear strands of ribonucleotides. Somewhat similarly to proteins, structured RNA can fold onto itself into one or even several competing three-dimensional (3D) structures. Given the biological and medical importance of RNA,predicting RNA tertiary structure from their respective nucleotide sequence would significantly boost related research.

After establishing the biological context of RNA, what can be done in terms of structure prediction for RNA? Unfortunately, there are many experimental wet-lab challenges^20^ when working with RNA, resulting in far fewer high-quality-related sequence and structural data than for proteins. Currently, we have only such data for about 70 non-redundant RNA families^21^ compared to >20,000 protein families^8^. This significant data gap makes the direct transfer of deep-learning approaches, such as AF2, to RNA not feasible (see below for more detail on deep-learning strategies). Yet despite the sparsity of data, first successes on RNA secondary structure prediction^22^ driven by machine learning (ML) and ML-derived improved scoring functions of RNA tertiary structure^23^ have been reported. Independent of these efforts and motivated by predicting RNA tertiary structure from their nucleotide sequence, predicting partial structural information in the form of spatial contacts is an established substitute procedure.

The statistical analysis of evolutionarily closely related sequences can (i) infer pairs of residues in spatial contact within biomolecules^24,25^ and (ii) guide the prediction of biomolecular structures when used in combination with molecular modeling techniques^26^. We focus on step (i) which is hampered by high false-positive rates or, more generally speaking, low signal-to-noise ratios. We use this intermediate to full-structure prediction as a proxy to evaluate self-supervised pre-training on RNA multiple sequence alignments (MSAs). Our full code (https://github.com/KIT-MBS/selbstaufsicht) and all model parameters (https://zenodo.org/record/8183962) are available under permissive open-source licenses.

Direct coupling analysis (DCA) is an established method for protein^27,28^ and RNA^24^ contact prediction based on sequence variability in the input MSAs^29,30^ via a Markov random field named generalized Potts model^31,32^. State-of-the-art methods use pseudo-likelihood maximization^33^ to fit the Potts model to an MSA. The DCA couplings are then ranked and interpreted as spatial contacts in the molecular structure. The major advantage of such methods is their unsupervised nature, i.e., that they do not need data annotation (labels) which is often cumbersome and time-consuming. Recent efforts in protein structure prediction have increasingly employed deep-learning strategies. As one of the most accessible types of data in this area is genetic sequences composed of monomers represented by letters, techniques popularized in natural language processing can be adapted to the biology domain. Since the last CASP competition for protein structure prediction^34^, the most successful methods^8,35^ harness the vast amounts of sequence and structure data^36–38^ to train large end-to-end^5^ transformer^39^ models, where DeepMind’s attention-based AF2 program is the most prominent example. AF2 iteratively refines a protein’s atomic coordinates predicted by its backbone and estimates its own error. This error estimation allows for a more efficient use of unlabeled sequence data by including high-confidence predictions of previously unlabeled samples in the training data.

Another way to utilize unlabeled data better is self-supervised learning. Self-supervised learning is not only used to enhance model performance with limited labeled data available^40^, but also to pre-train a model that can be adapted to a range of downstream tasks^41^. In self-supervised pre-training, the input data are augmented and the model is tasked with recovering information about the original input. Hence, the model is forced to learn patterns that shape its latent representations, which are then useful in a subsequent supervised downstream task.

Among the wide range of self-supervised tasks, common templates include masked language modeling^41^ or inpainting^42^, next-sentence prediction^41^ or jigsaws^43^, and contrastive learning^44^, all of which are established in language or image processing. Inpainting or hidden language modeling is one of the most ubiquitous self-supervised training tasks. A part of the information contained in the input sample is removed or distorted before passing through the model and has to be reconstructed from the context in the remaining sample. Jigsaw and next-sentence-prediction train the model to recover the relationship between fragments of a sample. Contrastive tasks use multiple, differently augmented views of the same sample to construct structural differences between samples. The model learns to identify whether or not two of these views originate from the same sample. Generative—or what we call bootstrapping—tasks create new samples or fragments using a generator, and a discriminator model has to distinguish between generated and original data.

For proteins, the ESM1b^45^ and MSA-Transformer^46^ models perform a masked language modeling task on single protein sequences and protein MSAs, respectively. As labeled data are much harder to come by for RNA than for proteins, establishing robust self-supervised models has the potential to close the gap in modeling quality between protein and RNA.

Contributors to the latest RNA-Puzzles round, a community-wide RNA structure prediction competition, still favored traditional energy-based approaches^47^ augmented by machine-learned contact prediction over purely ML-based approaches. ML for RNA structure is usually done in one of the following ways: (i) traditional DCA^24^, (ii) convolutional neural networks on single sequences^48^, and (iii) convolutional neural networks on MSA-/DCA-derived features^25,49^.

**Fig. 1:**
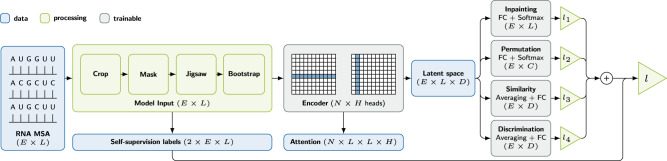
Model architecture for deep multi-task self-supervised pretext training to obtain RNA contact map candidates. Boxes represent model building blocks, edges information flow, and brackets tensor shapes. *L* is the length of the sequence, *E* the evolutionary dimension, i.e., available alignments, *N* the number of attention encoder blocks, *H* the number of attention heads, *C* the number of arrangements of the jigsaw, and *D* a parametric embedding dimension.
- evaluation of the impact of different upstream tasks on contact prediction performance,
- a weakly supervised RNA contact prediction model, we call BARNACLE,
- and publication of code and pre-trained model parameters.

Our contributions to RNA contact prediction in particular and the data sparsity often faced in ML applications for natural sciences in general are:

- Fine-tuning of a downstream decision tree model through a back-propagatable proxy model,
- self-supervised multi-task pre-training of an MSA transformer model for RNA (Fig. 1),

The dataset we use for downstream training and evaluation of our model is published (https://github.com/KIT-MBS/RNA-dataset.git)^21^ and was previously used to train the convolutional network CoCoNet^25^. It contains 57 structures in the training set and 23 structures in the test set. The upstream dataset of MSAs is taken from RFam^50^ which contains 4070 samples. We set aside 100 MSAs as an upstream validation set. A detailed description of all data used for training can be found in Supplementary Note I and in the respective references.

## Results

### Upstream

We first examine the upstream model in isolation. Table 1 shows upstream performance and training energy consumption. Since inpainting explicitly offers the model challenges at the single residue resolution and has already been shown to be a meaningful task, we use inpainting as the base task to be enhanced further with a secondary task. As the jigsaw, contrastive, and bootstrapping tasks are used only in conjunction with inpainting, we omit specifying the latter when combined with another task. Table 1 shows the self-supervised performance results for the model state with the lowest validation loss, where we measured the validation inpainting accuracy and the secondary task accuracy, if applicable. The total loss used is always the sum over all individual contributing task losses. A detailed description of the unsupervised tasks applied to MSAs, including augmentations and losses, can be found in Supplementary Note II. Detailed model descriptions are shown in Supplementary Tables I–IV. Training losses are shown in Supplementary Fig. 1. The model performs best on inpainting, when it does not have to divert resources to another task. Notably, we do not observe a synergy of tasks, where the training signal of one task improves performance on another.

**Table 1:** Pre-training (upstream) performance and energy consumption.

Task	Inpainting accuracy	Sec. task accuracy	Epochs	Energy/Wh
Inpainting	90.4%	—	2222	52,989.8
Jigsaw	83.1%	98.3 %	2264	53,837.6
Contrastive	89.9%	—	1707	50,540.8
Bootstrapping	83.1%	95.1 %	2182	52,874.2

We include energy measurements for each training run to put the resource footprint of our approach into perspective. A single pre-training run of 48 h consumes slightly over 50,000 Wh.

### Downstream

**Fig. 2:**
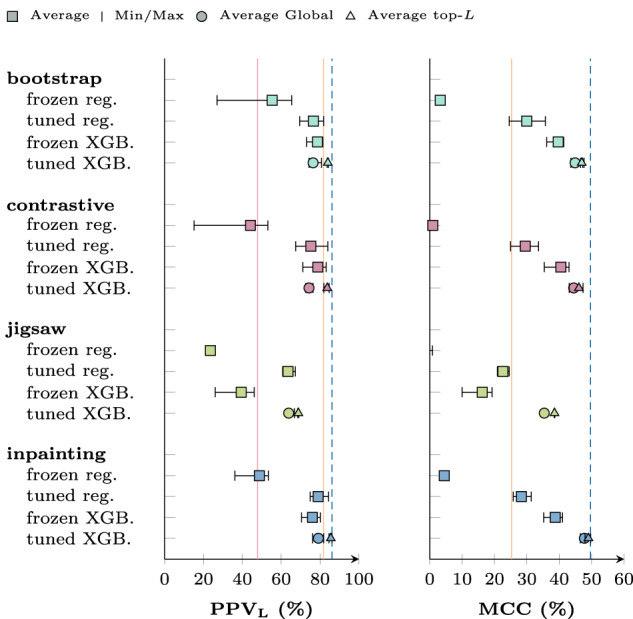
Downstream model performance of different unsupervised pre-training tasks and downstream training procedures in terms of top-*L* precision and global Matthews correlation coefficient on an independent test set. The red line shows the DCA baseline performance for PPV^21,24^, the orange line the shallow neural network CoCoNet^25^, and the dotted blue line the best-trained model performance. The square marker shows respective score averaged over several models trained with different early stopping metrics. Early stopping is performed using a small holdout set from the training dataset. The error bars show the best and worst score. For fine-tuned XGBoost we split top-*L* and global metrics used for backbone fine-tuning. One can directly observe an improvement of both PPV and MCC over the baseline in our approach.

Figure 2 shows the top-*L* PPV and Matthew’s correlation coefficient (MCC)^51^ for downstream contact prediction on the test dataset. Unless stated otherwise, we always report the micro-variant of a metric, i.e., we compute e.g., true positives over all samples and then compute the derived metric, instead of computing the metric for each sample and then averaging the metric. Top-*L*-PPV^25^, i.e., the precision measured only over the *L* most confident predictions in one sample assuming they are positive predictions, is a commonly used metric for contact prediction. Optimizing for this metric encourages the model to make at least *L* predictions. Furthermore, it has the benefit of being applicable to methods like mean-field DCA which produce rankings instead of interpretable scores. We also report MCC over all predictions to provide a global view and consider false negatives as well. In addition, Supplementary Table VI with the top-*L* precision with an untouched decision threshold of 0.5, as well as global precision, recall, F1-score, and the Matthews correlation coefficient, can be found in Supplementary Note VII.

The top-*L* precision for mean-field DCA averaged over the test dataset is 47.4%. This was improved to 81.4% by using a small convolutional network^25^ that filters out contacts based on the scores in its local environment. The MCC achieved by the baseline network is 25.28%. Using just the simple regression model with a frozen backbone, the equivalent of the MSA transformer on protein, yields models that have, at most, a slightly better top-*L* precision than the DCA baseline and sometimes below zero MCC scores.

Fine-tuning the backbone or switching to an XGBoost downstream model makes the models competitive with the baseline in terms of top-*L* precision and surpass it in MCC for most upstream tasks. Using XGBoost consistently has a larger impact on MCC, than fine-tuning the entire backbone model. The best models combine both of these modifications, i.e., training the XGBoost model with the attention maps generated by the fine-tuned backbone. These fine-tuned XGBoost models can beat CoCoNet in terms of top-*L* precision and almost double the MCC.

Other upstream tasks as additional pre-training signal do not have a positive impact on downstream performance. Adding neither contrastive nor bootstrap have a consistent and significant impact on the downstream performance compared to inpainting alone. Jigsaw, unfortunately degrades performance in particular for XGBoost models trained with the not fine-tuned backbone as input.

During downstream training, we build models for all the mentioned metrics through early stopping to prevent overfitting. Supplementary Section II contains more details, and Supplementary Fig. 2 shows the impact of the choice of early stopping metric on downstream performance. In Fig. 2, we show the scores for the best and worst model and the average over all of them. Fine-tuned XGBoost is treated slightly differently since the fine-tuning training is early-stopped with one metric and the XGBoost training itself with a potentially different one. We split top-*L* and global fine-tuning metrics (Loss, MCC and F1) because top-*L* metrics produce better performance, not just in top-*L* precision but also for global MCC. The early stopping metric of the XGBoost training itself has only limited impact.

Supplementary Fig. 3 in Supplementary Note V shows the importance of the attention map features in the downstream model. Supplementary Fig. 4 in Supplementary Note VI shows the impact MSA depth (i.e., the number of sequences in the MSA) has on downstream performance.

**Fig. 3:**
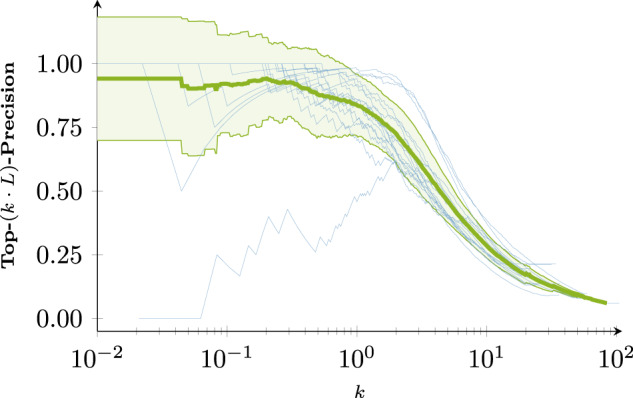
Macro-Top-(*k* ⋅ *L*)-precision. The light dotted lines show individual samples in the test set. The thick lines show the average. DCA, CoCoNet, and BARNACLE (tuned XGBoost) are shown in red, orange, and blue, respectively. The band around the average shows the standard deviation.

**Fig. 4:**
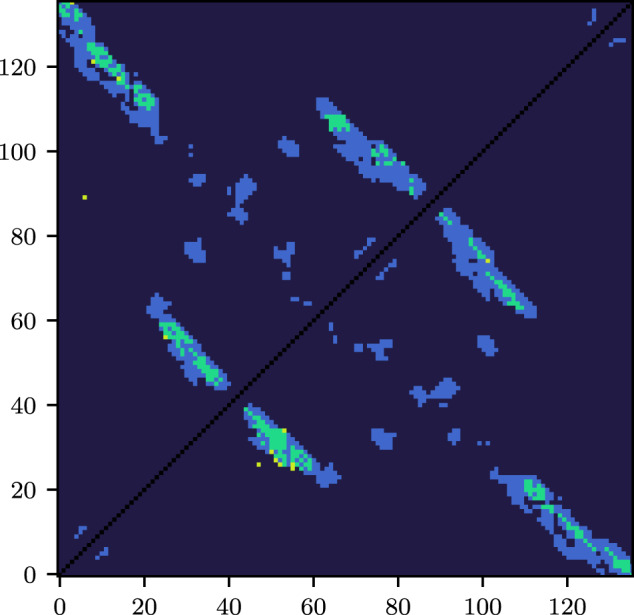
Contact map (PDB: 3ndb)—unfrozen vs. frozen. The upper left part shows the top-*L* contact predictions for the best model with unfrozen backbone, the lower right one for the best model with frozen backbone. Green pixels refer to true positives, yellow to false positives, light blue to false negatives, and dark blue to true negatives.

**Fig. 5:**
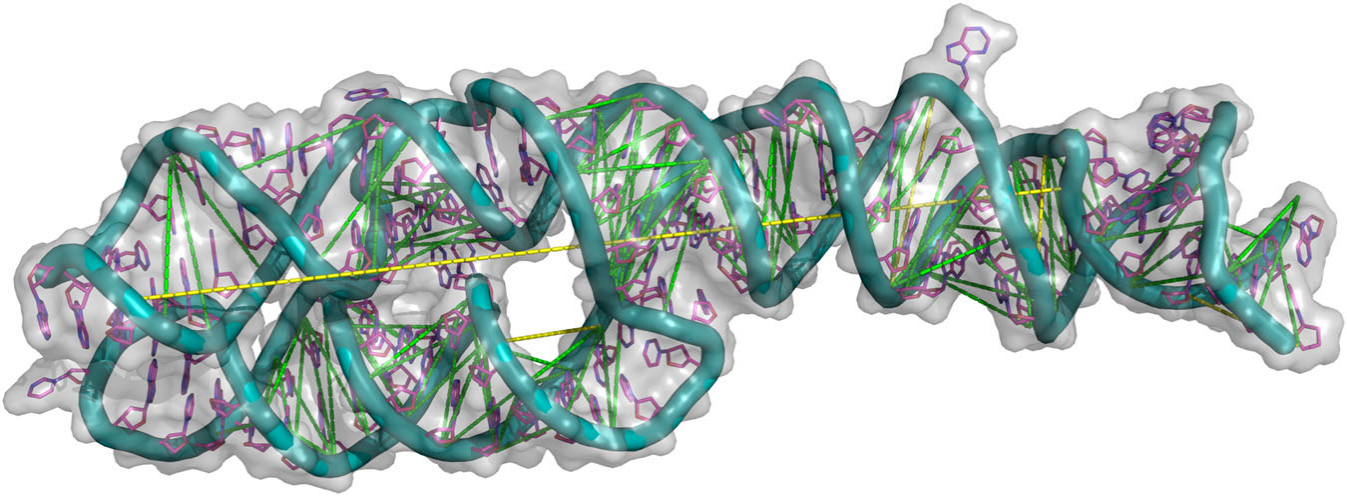
3D visualization of an RNA (PDB: 3ndb). Green dashed lines indicate correctly predicted inter-residue contacts, and yellow ones refer to false positives.

Figure 3 shows the precision computed over the most confident predictions as more contacts are included. Generally, the sharp decline begins just before *L* predictions are included. The most confident predictions of BARNACLE are more reliable than either baseline. Just before *L* predictions, precision begins to decline, with BARNACLE remaining in the lead. Figures 4 and 5 illustrate an example prediction from the test set. For this example, most of the false positives (with one exception) are only slightly above the threshold for the contact definition used. As they are close to a cluster of contacts in the contact map, they should not impede structure prediction. Supplementary Figs. 5 and 6 in Supplementary Note VIII show an additional, difficult example with more false positives. Closer analysis reveals that the specific RNA structure is one monomer of a dimer, with many false-positive contacts in the interaction pocket of the dimeric binding partner.

## Discussion

We demonstrated how to use self-supervised pre-training and XGBoost models suitable for sparse labeled data to gain more performance with limited data, despite these models not being end-to-end trainable. We explored the efficacy of our approach on the use case of RNA contact prediction and, in less depth, on RNA accessible surface area prediction (cf. Supplementary Note IV and Supplementary Table V).

Since the availability of MSA data for RNA is already limited, one complementary approach would be to train a larger language model on single sequences. This model would have to be larger because it has to encode the evolutionary relationships in its parameters, as they are no longer contained in the input data. The original motivation for using the attention maps for contact prediction in this way stems from the observation that they are correlated. Other techniques from the realm of explainable AI may be able to improve performance further or open new avenues to use the sparse existent data more efficiently.

As in other self-supervised pre-training settings, the bulk of the model can be adapted for other downstream applications to reduce from-scratch training. This is especially pertinent as monetary and environmental factors of training large models become more relevant. Our upstream hyperparameter search consumed upwards of 15 MW h.

We would like to stress that the choice of a target downstream metric is not a simple one. We hence report a wide range of metrics (Precision, F1, MCC) with top-*L* precision being the standard in the field. The exact choice of the downstream loss function (we tested focal loss, dice loss, cross-entropy, cf. Supplementary Note II) impacts these metrics slightly.

In conclusion, BARNACLE improves RNA contact prediction with limited labeled data. Such contacts can be used as restraints in tertiary structure prediction, or help to complement, interpret, or refine structural models based on measured experimental data from the wet-lab, such as incomplete NMR maps or data from low-resolution techniques such as small angle scattering. Considering the large gap between known RNA sequences and experimentally resolved tertiary structures, this represents a true breakthrough that significantly supports all structural RNA-related research by reducing the RNA sequence-structure gap and increase our knowledge about the genome’s “dark matter”. On a technical level, the BARNACLE approach might be a promising avenue for similar label starved fields in the natural sciences.

## Methods

### Pre-training

We employ different types of upstream tasks such as inpainting (or hidden language modeling), jigsaw, bootstrapping, and contrastive augmentations. Each one of the tasks is conceptually a self-supervised pretext task used to learn representations and extract patterns which are then exploited in the downstream task. In general, not every possible task is compatible with any other task in the sense that one task’s augmentation might impede another’s. For example, an auto-regressive task like next-token prediction and a task requiring a global view like a jigsaw would be difficult to train simultaneously.

#### Inpainting

Inpainting here is closer to masked language modeling, in that it is a classification task. The basic principle of masking tokens and recovering them can be implemented in a range of variations. The most important hyperparameters include the fraction of all tokens to mask, which tokens should replace the masked real tokens, and the shape of the mask (e.g., independent tokens or adjacent columns of tokens). We compute the classification loss over all masked tokens and experimented with different masking schemes. For our final model, randomly sampled individual tokens are replaced with a random legal token. As in the protein case, masking columns of the MSA does not increase performance. A more difficult version of the task might sample the replacement token from the MSA profile instead of uniformly.

We observe (cf. Supplementary Note II), that inpainting pre-training converges very slowly and inefficiently. We assume this is due to the nature of the random mask generation. The average sample generated during pre-training is likely not very informative for the model. Since implementing a mechanism akin to (semi-)hard example mining is difficult without more preexisting information, this remains a challenge.

#### Jigsaw

Each sequence in the MSA is split into a number of chunks which are then shuffled according to the assigned permutation. In this type of upstream task, permutations can be applied per sequence or, alternatively, the same permutation can be applied to the whole MSA. Other hyperparameters are the number of chunks and the number of permitted permutations. The model ultimately predicts the applied permutation in the sense of multi-class classification. The jigsaw upstream task in particular converges faster, but does not increase downstream performance, at least in the space we explored.

#### Bootstrapping

Here, parts of the MSA are bootstrapped according to the position-wise frequency distributions of tokens in the evolutionary dimension. Two variations are implemented: The first one randomly replaces whole sequences by synthetically generated sequences, the second one operates on token level. In both cases, the fraction of replaced sequences/tokens is an additional hyperparameter. In the end, the model has to determine which sequences/tokens are original and which were replaced, i.e., solve a binary classification problem.

#### Contrastive

Opposite to the other upstream tasks, the contrastive task does not involve any augmentation. Instead, the model generates one latent vector for each sequence in each MSA in the current batch. We then maximize the cosine similarity between sequences in the same MSA and minimize it between sequences from different MSAs.

### Downstream training

We employ two different types of models for downstream contact prediction. Both take the attention maps that are generated in a forward pass through the pre-trained backbone as an input and predict a contact score for each pair position in the map. This means, the input of a downstream model is a vector representing a single pixel in the contact map. The vector’s elements are collected from the respective pixel in all heads from all attention heads before the softmax is applied. The first model is a simple pixel-wise regression similar to the ‘unsupervised contact prediction’ in ref. ^46^. The second model uses boosted decision trees.

Apart from that, we differentiate between frozen and fine-tuned versions of both downstream models. For the frozen versions, the backbone parameters learned during pre-training are fixed, and only the parameters of the added downstream models are optimized. The fine-tuned regression models, by contrast, are optimized with respect to all parameters, including the backbone parameters. Last, the fine-tuned XGBoost model uses the re-trained backbone of the fine-tuned regression model and subsequently fits the decision trees.

#### Logistic regression

We use a classical logistic regression approach, where we model the log-odds of a residue pair being in contact as a linear combination of the corresponding attention map entries. Subsequently, the log-odds are transformed into contact probabilities by applying the sigmoid function. Contrary to traditional logistic regression, the bias parameter is omitted. This is due to the assumption that the log-odds should be represented only as a weighted sum of the corresponding attention values.

#### Boosted decision trees

Boosted decision trees are an ensemble model assembled by multiple weak *classification and regression trees* (CARTs). In comparison to a traditional decision tree, a CART assigns a real-valued score instead of a binary decision result to each leaf. Therefore, each CART *f* with *L* leaves and leaf scores ${{{{{{{\bf{w}}}}}}}}\in {{\mathbb{R}}}^{L}$ can be considered as a function *f*(**x**) = **w**_*q*(**x**)_, where ${{{{{{{\bf{x}}}}}}}}\in {{\mathbb{R}}}^{d}$ is a vector-valued input variable and $q:{{\mathbb{R}}}^{d}\to \{1,2,\ldots ,L\}$ models the tree structure. The ensemble model *F*_*K*_ consisting of *K* CARTs then sums up the scores corresponding to the leaves a given input **x** falls in, yielding the final model score ${F}_{K}({{{{{{{\bf{x}}}}}}}})=\mathop{\sum }\nolimits_{k = 1}^{K}{f}_{k}({{{{{{{\bf{x}}}}}}}})$. For the binary classification problem at hand, the model score is interpreted as log-odds representation, i.e., the actual classification result can be obtained by applying the sigmoid function, just as in logistic regression.

The ensemble model, *F*_*K*_, is built iteratively, adding a new tree per iteration. In turn, each newly added tree, *f*_*k*_, is structured in a way such that it reduces the deficiency of the previous model, *F*_*k*−1_, with respect to the given training data, as measured by some differentiable loss function. The results presented in this work were achieved using the XGBoost^52^ implementation of gradient-boosted trees. For more details, we refer the reader to its documentation.

#### Fine-tuning

To fine-tune the pre-trained backbone to a downstream task and data, we use the regression downstream model like we would for normal downstream training, keeping the backbone parameters unfrozen. We use early stopping with respect to all the relevant downstream metrics to avoid overfitting. The fine-tuned regression model is complete at this point. Since the XGBoost model can not be trained end-to-end with the backbone, we choose one of the early-stopped regression fine-tuning checkpoints as feature extractor for this new downstream model.

### Model architecture

Figure 1 shows the architecture of our model during pre-training.

Before passing the input MSA to the neural network, a pre-processing stage applies cropping and subsampling as well as augmentations and generates self-supervised labels.

After this pre-processing, the MSA is fed into a sequence of attention blocks, which represent the heart of the deep-learning model (also referred to as backbone). Each attention block is divided into several heads, each operating on only a subset of the incoming data, and performs tied axial attention on it similar to the MSA transformer^46^. The two most significant differences to this model are the usage of dropout layers subsequent to the attention operations and the replacement of the concluding single linear layer by a two-layered multilayer perceptron (MLP) with ReLU activation. The output of the final attention block is passed to the task heads. These consist for the most part of a reduction operation as task appropriate and a single linear layer and activation. We run one upstream training on a single node with four A100 GPUs over 48 h. In the current implementation, at least two GPUs are required for the contrastive task as the inter-MSA sequence distances are computed between GPU local batches.

For the downstream contact prediction training, we concatenate the latent attention maps instead of using the final latent output of the model as input for the contact task head. The contact task head consists of either the logistic regression or XGBoost model described earlier.

For more information on the hyperparameters and the training procedure, we refer to Supplementary Note III.

### High-performance computing environment

The experiments were run on the tier-2 high-performance computing system “Hochleistungsrechner Karlsruhe” (HoreKa) located at the Steinbuch Centre for Computing (SCC), Karlsruhe Institute of Technology. HoreKa is a distributed-memory, parallel hybrid supercomputer with nearly 60,000 Intel Xeon Ice Lake Scalable Processor cores, 220 TB main memory, and 668 NVIDIA A100 Tensor Core GPUs. HoreKa’s 769 compute nodes comprise 570 standard nodes, 32 high-memory nodes, and 167 accelerator nodes, each equipped with two 38-core Intel Xeon Platinum 8368 processors at 2.4 GHz base and 3.4 GHz maximum turbo frequency, 256 GB (standard) or 512 GB (high-memory and accelerator) local memory, a local 960 GB NVMe SSD disk, and two network adapters. The accelerator nodes have 4 NVIDIA A100-40 GPUs with 40 GB memory each. A low-latency, non-blocking NVIDIA Mellanox InfiniBand 4X HDR interconnect with 200 Gbit/s per port is used for communication between the nodes. The operating system installed on every node is Red Hat Enterprise Linux 8.2. HoreKa integrates the Helmholtz AI computing resources (HAICORE) partition with twelve GPU4 and three GPU8 accelerator nodes. A GPU4 node consists of 76 Intel Xeon Platinum 8368 processors, 512 GB main memory, four NVIDIA A100-40 GPUs, and a local 960 GB NVMe SSD disk. Each GPU8 node has 128 AMD “Rome” EPYC 7742 processors, 1 TB main memory, eight NVIDIA A100-40 GPUs, and six local NVMe SSD disks. We used Python v3.8 with biopython v1.79, numpy v1.20.3, torch v1.9.1.+cu111, torchmetrics v0.6.0, pytorch-lightning v1.5.1, and lightning-bolts v0.4.0.

### Reporting summary

Further information on research design is available in the Nature Portfolio Reporting Summary linked to this article.

### Supplementary information

Supplementary Notes

Description of Additional Supplementary Files

Supplementary Data Figure 2

Supplementary Data Figure 3

Reporting Summary

## Data Availability

Our full code (https://github.com/KIT-MBS/selbstaufsicht.git) are available under permissive open-source licenses. Source data for Figs. 2 and 3 can be found in Supplementary Data Fig. 2 and Supplementary Data Fig. 3.

## Code availability

Model parameters are available at (https://zenodo.org/record/8183962). All other data are available from the corresponding authors upon request.

## References

[1] Baker D, Sali A (2001). Protein structure prediction and structural genomics. Science.

[2] Schug A, Weigt M, Onuchic JN, Hwa T, Szurmant H (2009). High-resolution protein complexes from integrating genomic information with molecular simulation. Proc. Natl. Acad. Sci. USA.

[3] Hopf TA (2012). Three-dimensional structures of membrane proteins from genomic sequencing. Cell.

[4] Kamisetty H, Ovchinnikov S, Baker D (2013). Assessing the utility of coevolution-based residue–residue contact predictions in a sequence-and structure-rich era. Proc. Natl. Acad. Sci. USA.

[5] AlQuraishi M (2019). End-to-end differentiable learning of protein structure. Cell Syst..

[6] Calonaci N, Jones A, Cuturello F, Sattler M, Bussi G (2020). Machine learning a model for RNA structure prediction. NAR Genomics Bioinforma..

[7] Weiel M (2021). Dynamic particle swarm optimization of biomolecular simulation parameters with flexible objective functions. Nat. Mach. Intell..

[8] Jumper J (2021). Highly accurate protein structure prediction with AlphaFold. Nature.

[9] Chowdhury, R. et al. Single-sequence protein structure prediction using a language model and deep learning. *Nat. Biotechnol.* **40**, 1617–1623 (2022). 10.1038/s41587-022-01432-w PMC10440047 36192636

[10] Anishchenko I (2021). De novo protein design by deep network hallucination. Nature.

[11] Varadi M (2022). AlphaFold Protein Structure Database: massively expanding the structural coverage of protein-sequence space with high-accuracy models. Nucleic Acids Res..

[12] Bryant P (2022). Predicting the structure of large protein complexes using AlphaFold and Monte Carlo tree search. Nat. Commun..

[13] Chi KR (2016). The dark side of the human genome. Nature.

[14] Gilbert, W. Origin of life: The RNA world. *Nature* **319**, 618 (1986).

[15] Eddy SR (2014). Computational analysis of conserved RNA secondary structure in transcriptomes and genomes. Annu. Rev. Biophys..

[16] Zhao EM (2022). RNA-responsive elements for eukaryotic translational control. Nat. Biotechnol..

[17] Dolgin E (2019). Unlocking the potential of vaccines built on messenger RNA. Nature.

[18] Jackson, L. A. et al. An mRNA vaccine against SARS-CoV-2—preliminary report. *New Engl. J. Med.* **383**, 1920–1931 (2020). 10.1056/NEJMoa2022483 PMC7377258 32663912

[19] Mulligan MJ (2020). PhaseI/II study of COVID-19 RNA vaccine BNT162b1 in adults. Nature.

[20] Zhang J, Fei Y, Sun L, Zhang QC (2022). Advances and opportunities in RNA structure experimental determination and computational modeling. Nat. Methods.

[21] Pucci F, Zerihun MB, Peter EK, Schug A (2020). Evaluating DCA-based method performances for RNA contact prediction by a well-curated data set. RNA.

[22] Sato K, Akiyama M, Sakakibara Y (2021). RNA secondary structure prediction using deep learning with thermodynamic integration. Nat. Commun..

[23] Townshend RJ (2021). Geometric deep learning of RNA structure. Science.

[24] DeLeonardis E (2015). Direct-Coupling Analysis of nucleotide coevolution facilitates RNA secondary and tertiary structure prediction. Nucleic Acids Res..

[25] Zerihun MB, Pucci F, Schug A (2021). CoCoNet—boosting RNA contact prediction by convolutional neural networks. Nucleic Acids Res..

[26] Boniecki MJ (2016). SimRNA: a coarse-grained method for RNA folding simulations and 3D structure prediction. Nucleic Acids Res..

[27] Weigt M, White RA, Szurmant H, Hoch JA, Hwa T (2009). Identification of direct residue contacts in protein–protein interaction by message passing. Proc. Natl. Acad. Sci. USA.

[28] Morcos F (2011). Direct-coupling analysis of residue coevolution captures native contacts across many protein families. Proc. Natl. Acad. Sci. USA.

[29] Eddy SR (2011). Accelerated profile HMM searches. PLoS Comput. Biol..

[30] Nawrocki EP, Eddy SR (2013). Infernal 1.1: 100-fold faster RNA homology searches. Bioinformatics.

[31] Wu FY (1982). The Potts model. Rev. Mod. Phys..

[32] Nguyen HC, Zecchina R, Berg J (2017). Inverse statistical problems: from the inverse Ising problem to data science. Adv. Phys..

[33] Ekeberg M, Lövkvist C, Lan Y, Weigt M, Aurell E (2013). Improved contact prediction in proteins: using pseudolikelihoods to infer Potts models. Phys. Rev. E.

[34] Pereira J (2021). High-accuracy protein structure prediction in CASP14. Proteins Struct. Funct. Bioinforma..

[35] Baek M (2021). Accurate prediction of protein structures and interactions using a three-track neural network. Science.

[36] Berman HM (2000). The protein data bank. Nucleic Acids Res..

[37] Mirdita M (2017). Uniclust databases of clustered and deeply annotated protein sequences and alignments. Nucleic Acids Res..

[38] Steinegger M, Mirdita M, Söding J (2019). Protein-level assembly increases protein sequence recovery from metagenomic samples manyfold. Nat. Methods.

[39] Vaswani, A. et al. Attention is all you need. *Adv. Neural Inf. Process. Syst.* **30** https://proceedings.neurips.cc/paper/2017/hash/3f5ee243547dee91fbd053c1c4a845aa-Abstract.html (2017).

[40] Doersch, C., Gupta, A. & Efros, A. A. Unsupervised Visual Representation Learning by Context Prediction. in *2015 IEEE International Conference on Computer Vision (ICCV)*, 1422–1430 (IEEE, 2015).

[41] Devlin, J., Chang, M.-W., Lee, K. & Toutanova, K. BERT: Pre-training of Deep Bidirectional Transformers for Language Understanding. in *Proceedings of the 2019 Conference of the North American Chapter of the Association for Computational Linguistics: Human Language Technologies, Volume 1 (Long and Short Papers)*, 4171–4186 (Association for Computational Linguistics, Minneapolis, Minnesota, 2019).

[42] Pathak, D., Krahenbuhl, P., Donahue, J., Darrell, T. & Efros, A. A. Context encoders: Feature learning by inpainting. in *Proceedings of the IEEE Conference on Computer Vision and Pattern Recognition*, 2536–2544 (IEEE, 2016).

[43] Noroozi, M. & Favaro, P. Unsupervised learning of visual representations by solving jigsaw puzzles. in *European Conference on Computer Vision*, 69–84 (Springer, 2016).

[44] Chen, T., Kornblith, S., Norouzi, M. & Hinton, G. A simple framework for contrastive learning of visual representations. in *Proceedings of the 37th International Conference on Machine Learning*, 1597–1607 (PMLR, 2020).

[45] Rives, A. et al. Biological structure and function emerge from scaling unsupervised learning to 250 million protein sequences. *Proc. Natl. Acad. Sci. USA* **118**, e2016239118 (2021). 10.1073/pnas.2016239118 PMC8053943 33876751

[46] Rao, R. M. et al. MSA Transformer. in *Proceedings of the 38th International Conference on Machine Learning*, *Vol. 139 of**Proceedings of Machine Learning Research*, 8844–8856 (PMLR, 2021).

[47] Miao Z (2020). RNA-Puzzles Round IV: 3D structure predictions of four ribozymes and two aptamers. RNA.

[48] Singh J, Hanson J, Paliwal K, Zhou Y (2019). RNA secondary structure prediction using an ensemble of two-dimensional deep neural networks and transfer learning. Nat. Commun..

[49] Singh J (2021). Improved RNA secondary structure and tertiary base-pairing prediction using evolutionary profile, mutational coupling and two-dimensional transfer learning. Bioinformatics.

[50] Kalvari I (2021). Rfam 14: expanded coverage of metagenomic, viral and microRNA families. Nucleic Acids Res..

[51] Matthews BW (1975). Comparison of the predicted and observed secondary structure of T4 phage lysozyme. Biochimica et. Biophysica Acta (BBA)-Protein Struct..

[52] Chen, T. & Guestrin, C. XGBoost: a scalable tree boosting system. in *Proceedings of the 22nd ACM SIGKDD International Conference on Knowledge Discovery and Data Mining*, KDD ’16, 785–794 (ACM, 2016).

