# Supplementary material for: RNA contact prediction by data efficient deep learning
Source: Commun Biol. 2023 Sep 6;6:913. doi: 10.1038/s42003-023-05244-9 (PMC10482910; doi:10.1038/s42003-023-05244-9)
Supplement: Supplementary file 1 — Supplementary Notes [file 42003_2023_5244_MOESM1_ESM.pdf]

RNA Contact Prediction by Data Efficient Deep Learning (Supplementary Notes)

Oskar Taubert,[1] Fabrice von der Lehr,[2] Alina Bazarova,[3,4] Christian Faber,[3]
Philipp Knechtges,[2] Marie Weiel,[1,4] Charlotte Debus,[1,4] Daniel Coquelin,[1,4] Achim Basermann,[2]
Achim Streit,[1] Stefan Kesselheim,[3,4] Markus Götz,[1,4,*] and Alexander Schug[3,5,*]

[1]*Steinbuch Centre for Computing, Karlsruhe Institute of Technology, 76344 Eggenstein-Leopoldshafen, Germany*
[2]*Institute for Software Technology (SC), German Aerospace Centre (DLR), 51147 Köln, Germany*
[3]*Jülich Supercomputing Centre, Forschungszentrum Jülich, 52428 Jülich, Germany*
[4]*Helmholtz AI*
[5]*Faculty of Biology, University of Duisburg-Essen, 45117 Essen, Germany*

(Dated: August 11, 2023)

SUPPLEMENTARY NOTE I. DATASETS

Rfam 14.6 [1, 2] is a database of RNA families, each comprising a multiple sequence alignment, consensus secondary structure and a covariance model. Seed alignments of Rfam dataset contain manually curated set of the most representative sequences of the family, whilst the full ones comprise all known members. For each available MSA in Rfam we use full alignment only in case the corresponding seed alignment consists of less than 100 sequences. Overall there are 4070 MSAs each comprising between 10 and 5000 sequences.

Zasha Weinberg Data [3] are the alignments available on `https://bitbucket.org/zashaw/zashaweinbergdata/src/master/`. We use 43 MSAs which were not previously submitted to Rfam database with numbers of sequences ranging between 8 and 650. We combine these as our pre-training dataset. For cross-validation we hold back a random set of MSAs from the combined set.

The dataset used in [4] is based on the one presented in [5]. The latter one was curated by increasing E-value cutoff of the Rfam 14.1 RNA families to 0.99 and using the cmsearch option of Infernal [6] without modifying covariance models. Out of 70 RNA structures with high resolution 57 were selected thereafter and used as a training set. Additional 23 RNA structures were collected by relaxing the structure resolution criteria of [5] and used as a test set. Number of sequences in the MSAs ranges between 6 and 190000. For model validation and hyperparameter search during downstream training we separate a 20% split of the structure training data.

SUPPLEMENTARY NOTE II. TRAINING DETAILS

A. Loss Functions

In general, all our upstream and the downstream task are modeled as classification problems. We therefore use different variations of cross-entropy loss to train our models: Inpainting and Jigsaw employ categorical cross-entropy, Bootstrapping uses binary cross-entropy, and normalized temperature-scaled cross-entropy loss (NT-Xent loss, [7]) is applied to the Contrastive task. In the downstream training of the regression model, we use focal loss [8] with parameters $\alpha = 0.95, \gamma = 2$ to counteract the class imbalance between contacts and non-contacts. We also tested regular cross-entropy and dice loss [9], but found that these perform worse.

In case of a conjunction of several upstream tasks, we define the total loss $\mathcal{L}$ used for training as unweighted sum of the individual task-specific losses

$$\mathcal{L} = \sum_{t \in \mathcal{T}} \mathcal{L}_t, \tag{1}$$

where $\mathcal{T} \subseteq \{\text{inpainting}, \text{jigsaw}, \text{contrastive}, \text{bootstrapping}\}$ denotes the set of tasks being combined in the training of a specific upstream model and $\mathcal{L}_{\text{inpainting}}$, $\mathcal{L}_{\text{jigsaw}}$, $\mathcal{L}_{\text{bootstrapping}}$, and $\mathcal{L}_{\text{contrastive}}$ denote the corresponding individual task losses. We also tested a weighted sum, but found that these additional parameters did not have meaningful impact. Figure 1 shows the loss progression over the course of pre-training. Note that the losses are mostly in the same order of magnitude. Contrastive divergres the most from inpainting, but still does not dominate the training signal.

B. Training Parameters

Table I summarizes the parametrization of our deep-learning-based models.

* Joint corresponding authors.

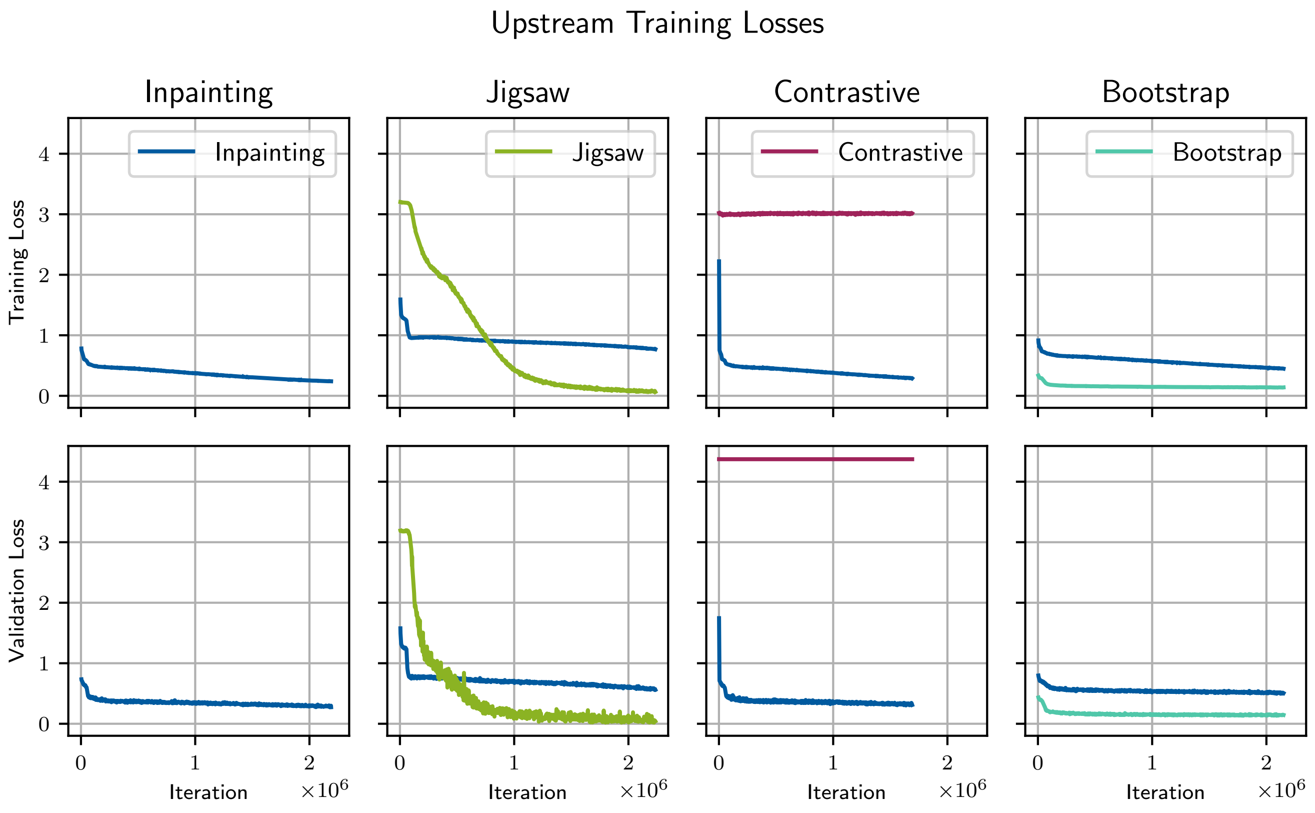

SUPPLEMENTARY FIGURE 1. Upstream loss components over the course of pre-training.

Pre-training Parameters

The upstream model consists of 10 attention blocks with 12 heads and 64 features per head, totalling almost 60M parameters. We use a local batch size of 1 and train each upstream model for 2 days. Adam is employed as optimization algorithm. The learning rate is set to 3×10^{-5} with an linear warm-up phase of 400 epochs and a subsequent decay according to an inverse square root rule. To increase the training speed, we use 16-bit mixed precision. Furthermore, the dropout layers in the attention blocks operate with a drop-out ratio of 0.3. In order to stay within the memory limits of our system, we crop and subsample large MSAs randomly to sequence length 400 and sequence count 50, respectively. Performance metrics (e.g., accuracy) are evaluated on a random subset of 100 MSAs excluded from the training dataset.

Inpainting is performed token-wise, with a masking ratio of 0.15. Masked tokens are replaced by a random token from the set of regular sequence tokens. For Jigsaw, we divide sequences into 4 chunks and allow all 24 possible permutations. Similar to Inpainting, the token-wise implementation of the Bootstrapping task has also proven to be best. Here, however, we use a replacement ratio of 0.5. Other values did not lead to significant improvement in the upstream performance. Last, the temperature in the Contrastive task is set to 100.

Downstream Training Parameters

While architectural parameters of the backbone remain the same, we adapt some of the training parameters compared to the pre-training. For example, since the labeled training dataset is drastically smaller than the unlabeled dataset used for pre-training, we are able to use diversity-maximizing subsampling as in [11]. Moreover, a random selection of 20% of the training data samples is withheld from training and used as a validation dataset.

The regression model is trained for at most 30 minutes, but we early stop w.r.t. loss or the performance metrics top-$L_{0.5}$-precision, top-L-precision, F1 score, and Matthews correlation coefficient on the validation data. Both dropout and learning rate scheduling are disabled, and the learning rate is set to 1×10^{-4}. These settings apply to both the training of the actual regression model and the re-training of the backbone for the finetuned XGBoost model.

Table II contains the hyperparameters of the XGBoost models. Every XGBoost model consists of at most 300 trees with maximum depth 16. It is built using gradient-based subsampling, where the subsampling rate is set to 0.9. Again, we perform early stopping w.r.t. to

SUPPLEMENTARY TABLE I. Pre-training and finetuning parameters of the deep-learning-based models.

**Parameter**	**Pre-training**	**Finetuning**
Architecture		
# Attention blocks	10	10
# Heads per block	12	12
# Features per head	64	64
Batch size (local)	1	1
Optimizer	Adam	Adam
Learning rate	3×10^{-5}	1×10^{-4}
Warm-up	linear, 400 epochs	–
Decay	inverse square root, after 400 epochs	–
Drop-out ratio	0.3	0
Pre-processing		
Cropping mode	random	random
Cropping size	400	400
Subsampling mode	random	diversity-maximizing
Subsampling size	50	50
Inpainting		
Mode	token-wise	–
Masking ratio	0.15	–
Replacing tokens	regular seq. tokens	–
Jigsaw		
# Chunks	4	–
# Permutations	24	–
Bootstrapping		
Mode	token-wise	–
Replacement ratio	0.5	–
Contrastive		
Temperature	100	–
Validation data size	100 MSAs	20%
Float precision	16-bit mixed	16-bit mixed
Training time	2 days	$<$ 30 minutes (incl. early stopping)

SUPPLEMENTARY TABLE II. Training parameters of the XGBoost models. Other parameters are set to their default values.

**Parameter**	**Value**
# Trees (max)	300
Tree depth (max)	16
Learning rate	1.0
Booster	DART [10]
Drop-out ratio	0.1
Subsampling	
Mode	gradient-based
Rate	0.9
Colsample	
By-tree	0.7
By-level	0.7
Minimum split loss	0.7
Objective	binary:logitraw
Tree method	gpu_hist
Training time	$<$ 10 minutes (incl. early stopping)

the above metrics on the validation data. We also employ a dropout-like extension called DART [10] with drop-out rate 0.1. Aside from that, we use the following parametrization: learning-rate 1.0, minimum-split-loss 0.7, minimum-child-weight 1.0, colsample-bytree 0.7, colsample-bylevel 0.7.

C. Finetuning Monitor Metrics

Figure 2 illustrates the impact the choice of early stopping metric used during finetuning has on the final model performance. We stop training the model, once the chosen metric begins to degrade on the validation set. The monitor metric of the XGBoost training itself has only negligible impact, but the top-L style metrics seem to force the backbone to produce features that are more useful for XGBoost contact predictor model. Using global metrics instead produces worse XGBoost models. Especially in the contrastive case, using $F1$-score as the XGBoost monitor metric, seems to remove this advantage again. Notably, always using e.g. MCC for both finetuning early-stopping and XGBoost monitor metric does not produce the highest MCC score for the final model.

D. Parallelization

We employ data-parallelism to further speed-up our pre-training by distributing the load to four GPUs. The downstream training, however, is conducted only on one GPU. For the XGBoost model, this is because its fitting cannot be data-parallelized. For the regression model, on the other hand, we do not run into training time issues as for pre-training, so we rather aim to keep the effective batch size minimal in order to achieve the maximum effect of stochastic gradient descent.

The parallelization strategy used for pre-training works as follows: First, before the actual training is started, the training dataset is distributed to the allocated GPUs, i.e., each GPU obtains visibility only into a subset of the overall dataset. During training, gradient steps are synchronized: All GPUs perform a forward and backward pass and subsequently wait for each other. Then, the computed local gradients are collected, shared and averaged. In the end, weights on each GPU are updated by the same global gradient.

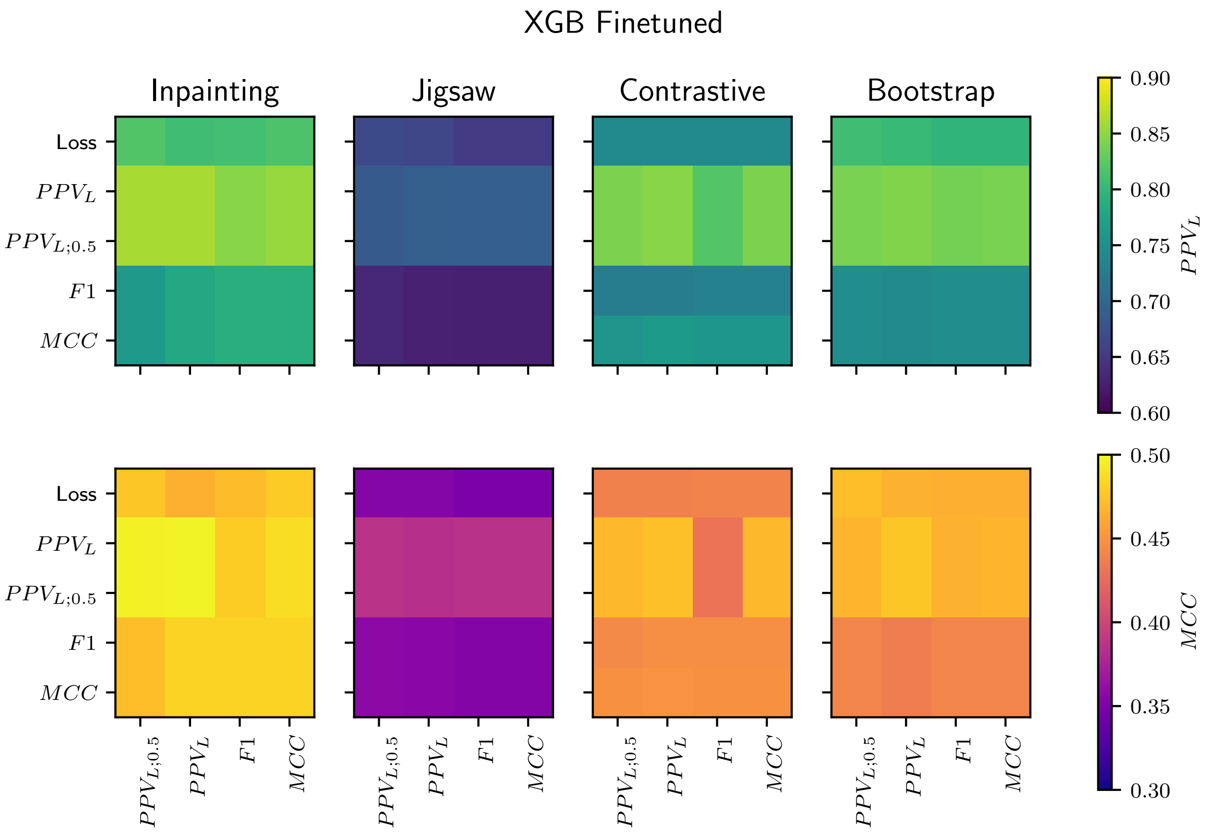

SUPPLEMENTARY FIGURE 2. Finetuned XGBoost performance metric heatmaps for the different combinations of early stopping metric used during the regression finetuning of the pre-trained model and the monitor metric of the XGBoost training. The top row shows top-L-precision, the bottom row MCC.

SUPPLEMENTARY NOTE III. HYPERPARAMETER SEARCH AND ABLATION STUDIES

We tune the hyperparameters for the upstream and the downstream separately. For the backbone we use a random search in the limits in table III. We run 300 iterations of this search and arrive at the parameters given in table I.

For the downstream regression model we only experimented with adding a hidden layer and adding biases, none of which showed any benefit.

SUPPLEMENTARY TABLE III. Parameter limits for the optimization of hyperparameters in the upstream training.

**Parameter**	**Limits**
# blocks	{6, 8, 10}
# heads	{8, 12, 16}
d_{head}	{16, 32, 64, 128}
learning rate	$[10^{-6}, 10^{3}]$
dropout	$[0, 0.5]$
Inpainting masking mode	{token, column}
Jigsaw partitions	{3, 4, 5}
Contrastive Temperature	$[10, 100]$

We use an evolutionary optimization[12] to explore the parameter space for the XGBoost models. Parameter limits are listed in table IV. We use 16 workers to evaluate individual candidate solutions in parallel for about 6000 generations. The population size is set to 16 as well, i.e., each worker operates on a single parametrization candidate. The algorithm to generate new individual candidates is a simple combination of cross-breeding, point mutation, and selection for top-L precision. Moreover, each of the 4 disjointed sets of 4 workers forms an island being isolated from the others with respect to cross-breeding. The probability for migration between those islands is set to 0.1. Since a single XGBoost training only runs for few minutes instead of days, the energy footprint of this search is negligible next to the unsupervised backbone training.

SUPPLEMENTARY TABLE IV. Parameter limits for the optimization of hyperparameters in the XGBoost training.

Parameter	Limits
# Trees (max)	$[1, 500]$
Tree depth (max)	$[4, 16]$
Learning rate	$[0.01, 1.0]$
Drop-out ratio	$[0, 0.5]$
Subsampling	
Mode	{uniform, gradient-based}
Rate	$[0.4, 1]$
Colsample	
By-tree	$[0.4, 1]$
By-level	$[0.4, 1]$
Minimum split loss	$[0, 1]$

SUPPLEMENTARY NOTE IV. GENERALIZABILITY

The general principle of using a deep neural network pre-trained on unlabeled data in a self-supervised fashion, that then serves as a feature extraction mechanism for a data efficient downstream model, is applicable to other tasks. To demonstrate this generalizability, we apply our approach to solvent accessibility surface area (ASA) prediction for RNA, a related problem to our primary task. Unlike contacts, ASA is regressed for each token in the target sequence. Instead of the latent attention maps of the upstream model, the downstream regression uses the latent output of the upstream model as input. We use the messenger RNA temperature adaptation dataset from [13] available at http://zhouyq-lab.szbl.ac.cn/download/, which consists of 172 MSAs, each merged into one file and aligned using the clustalw software [14] and subsequently split into a training (80%) and validation (20%) sets. Labels were extracted from the corresponding .asa files and the insertions were substituted by -1 values and the corresponding mask is applied to the predictions so that these values do not contribute to the evaluation procedure. In order to employ the previously pre-trained self-supervised models as a backbone, we crop MSAs longer than 400 bases to use only the middle 400 bases as input. We trained the downstream regression model for 100 epochs and report the pearson correlation between the prediction and the label ASA in table V. The pattern we found for contact prediction persists: XGB models benefit from the finetuned feature extraction, outperforming an end-to-end trained neural network. We did not perform an exhaustive hyperparameter search for this task, which has the potential to increase performance further. The best model we found, finetuned XGB with Inpainting, significantly beats the baseline of 0.63 reported in [13].

	Frozen NN	Tuned NN	Frozen XGB	Tuned XGB
Jigsaw	0.1731	0.4849	0.4877	0.6740
Contrastive	0.1832	0.5292	0.4930	0.7194
Bootstrap	0.1115	0.4938	0.4927	0.6984
Inpainting	0.1934	0.5222	0.4905	0.7443

SUPPLEMENTARY TABLE V. Pearson correlation coefficient for ASA prediction. The baseline performance is 0.63.

SUPPLEMENTARY NOTE V. INTERPRETATION OF ATTENTION MAPS

Figure 3 illustrated the relative importance for the downstream input feature maps originating from individual attention heads. The regression model using a frozen backbone focuses more on specific and on earlier heads. The finetuning spread the focus of the model over evenly over mostly deeper heads. The XGBoost model focuses already on later heads without finetuning. Using features from the finetuned backbone as input for the XGBoost model focuses it on heads of intermediate depth, but does not make it as diffuse as the finetuned regression.

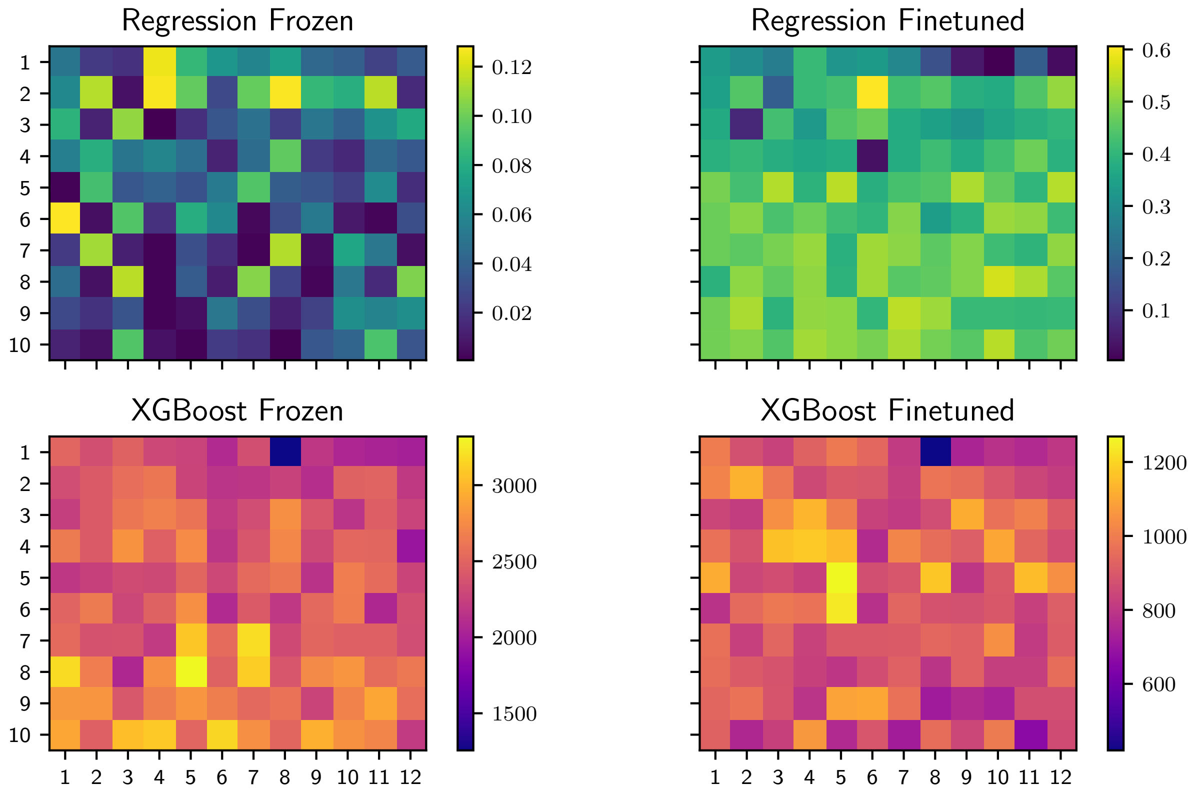

SUPPLEMENTARY FIGURE 3. Absolute values of the regression model parameters (top) and XGBoost feature importance scores (bottom) for a selection of downstream models with fixed (left) and finetuned (right) backbone, respectively. The regression models are pre-trained with Bootstrapping and optimized for global F1 score. The XGBoost models are pre-trained with just Inpainting and optimized for top-L precision.

SUPPLEMENTARY NOTE VI. MSA DEPTH

Figure 4 shows the performance of BARNACLE (inpainting finetuned XGBoost) and the baselines over the number of sequences in the alignments in the test dataset. Note that BARNACLE is at a slight disadvantage here, because we sample only 50 sequences from the alignment, albeit while maximizing their diversity. Nonetheless, it is expected, that the performance of all the shown models deteriorates as MSAs become too shallow to contain relevant evolutionary information.

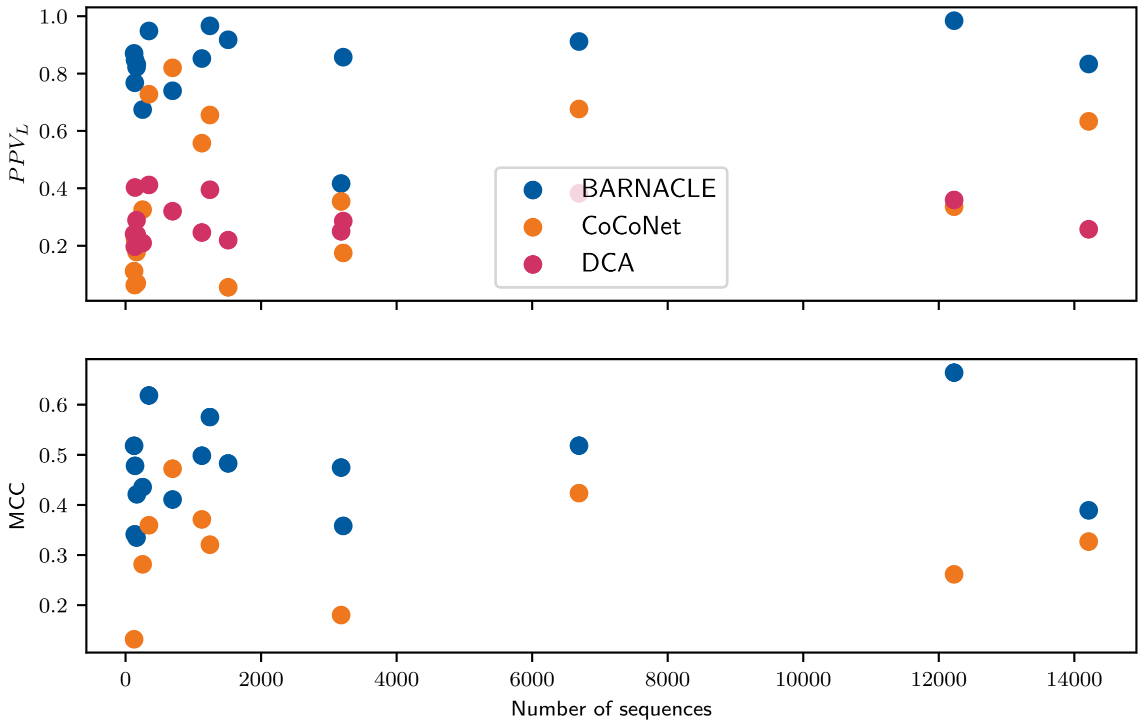

SUPPLEMENTARY FIGURE 4. Performance over number of sequences in the alignment.

SUPPLEMENTARY NOTE VII. EXTENDED RESULTS TABLE

Table VI shows all measured metrics over all models that were only shown in aggregate in the main paper.

SUPPLEMENTARY TABLE VI: Extended results

contacthead frozen							
inpainting Loss	53.44	53.44	8.82	100.00	16.21	nan	3.84
inpainting PPV_L	53.44	53.44	8.82	100.00	16.21	nan	3.59
inpainting $PPV_{L;0.5}$	53.44	53.44	8.82	100.00	16.21	nan	4.50
inpainting $F1$	47.33	47.33	9.05	97.62	16.57	3.59	3.57
inpainting MCC	36.16	36.16	10.31	59.92	17.59	5.37	4.74
jigsaw Loss	24.73	24.73	8.82	100.00	16.22	0.16	3.73
jigsaw PPV_L	22.93	22.93	8.44	77.57	15.22	-2.83	3.27
jigsaw $PPV_{L;0.5}$	22.93	22.93	8.44	77.57	15.22	-2.83	3.63
jigsaw $F1$	23.57	23.57	8.82	100.00	16.22	0.16	3.59
jigsaw MCC	23.70	23.70	8.83	99.98	16.23	0.81	3.76
contrastive Loss	53.11	53.11	8.82	100.00	16.21	nan	3.59
contrastive PPV_L	53.18	53.18	8.82	100.00	16.21	nan	3.71
contrastive $PPV_{L;0.5}$	53.18	53.18	8.82	100.00	16.21	nan	3.79
contrastive $F1$	46.31	46.31	8.93	98.55	16.38	2.36	3.65
contrastive MCC	15.16	15.16	8.68	50.96	14.84	-0.51	3.70
bootstrap Loss	65.45	65.45	8.82	100.00	16.21	nan	3.85
bootstrap PPV_L	65.38	65.38	8.82	100.00	16.21	nan	4.67
bootstrap $PPV_{L;0.5}$	65.38	65.38	8.82	100.00	16.21	nan	3.68
bootstrap $F1$	53.76	53.76	8.95	99.12	16.42	2.92	4.06
bootstrap MCC	26.97	26.97	10.15	42.32	16.37	3.56	4.08
contacthead finetuned							
inpainting Loss	74.95	74.95	19.96	73.90	31.43	27.35	3.83
inpainting PPV_L	84.33	84.33	19.23	72.02	30.36	25.78	5.49
inpainting $PPV_{L;0.5}$	84.33	84.33	19.23	72.02	30.36	25.78	4.54
inpainting $F1$	75.85	75.85	23.08	73.21	35.09	31.33	4.56
inpainting MCC	75.85	75.85	23.08	73.21	35.09	31.33	5.42
jigsaw Loss	61.34	61.34	16.50	75.10	27.05	22.17	4.28
jigsaw PPV_L	67.31	67.31	15.50	77.33	25.83	20.88	4.04
jigsaw $PPV_{L;0.5}$	67.31	67.31	15.50	77.33	25.83	20.88	4.93
jigsaw $F1$	60.76	60.76	19.34	66.85	30.00	24.56	4.88
jigsaw MCC	60.76	60.76	19.34	66.85	30.00	24.56	5.41
contrastive Loss	72.00	72.00	21.85	75.38	33.88	30.39	3.05
contrastive PPV_L	84.01	84.01	18.84	71.18	29.79	24.97	3.80
contrastive $PPV_{L;0.5}$	84.01	84.01	18.84	71.18	29.79	24.97	3.68
contrastive $F1$	67.50	67.50	26.35	67.90	37.96	33.52	3.65
contrastive MCC	68.85	68.85	26.15	68.82	37.90	33.59	3.80
bootstrap Loss	79.83	79.83	21.69	72.45	33.38	29.32	3.63
bootstrap PPV_L	81.89	81.89	18.52	71.38	29.41	24.54	3.72
bootstrap $PPV_{L;0.5}$	81.89	81.89	18.52	71.38	29.41	24.54	3.77
bootstrap $F1$	69.49	69.49	30.49	61.95	40.87	35.71	3.74
bootstrap MCC	69.49	69.49	30.49	61.95	40.87	35.71	3.63
xgb frozen							
inpainting $PPV_{L;0.5}$	83.72	79.90	78.71	24.08	36.88	41.04	3.90
inpainting PPV_L	84.39	80.15	78.80	23.99	36.78	40.99	4.79
inpainting $F1$	70.46	70.46	53.77	28.62	37.35	35.18	2.59
inpainting MCC	74.06	73.80	61.25	27.98	38.42	37.88	3.47
jigsaw $PPV_{L;0.5}$	87.08	43.93	87.08	4.78	9.07	19.26	3.81
jigsaw PPV_L	89.69	46.11	89.69	4.43	8.44	18.86	4.58
jigsaw $F1$	26.01	26.01	18.35	16.90	17.60	9.99	3.63
jigsaw MCC	63.01	41.62	63.01	5.49	10.11	16.82	3.78
contrastive $PPV_{L;0.5}$	85.46	83.17	77.26	26.53	39.50	42.65	4.12
contrastive PPV_L	84.58	82.02	77.69	26.83	39.88	43.04	3.74

Task + checkpoint metric(s)	$PPV_{L;0.5}$ / %	PPV_L / %	PPV / %	SEN / %	F1 / %	MCC / %	Energy / Wh
contrastive $F1$	71.16	71.16	50.70	31.16	38.60	35.36	2.77
contrastive MCC	79.84	79.13	67.14	29.05	40.55	40.96	4.60
bootstrap $PPV_{L;0.5}$	87.07	81.44	79.90	23.68	36.53	41.06	3.78
bootstrap PPV_L	86.29	80.92	79.65	24.11	37.01	41.37	3.34
bootstrap $F1$	73.43	73.09	56.12	28.54	37.84	36.14	3.17
bootstrap MCC	82.11	79.45	73.60	25.00	37.33	40.15	4.54
			xgb finetuned				
inpainting Loss $PPV_{L;0.5}$	81.89	81.89	57.13	47.15	51.67	47.73	3.87
inpainting Loss PPV_L	80.80	80.80	54.94	47.08	50.70	46.50	4.57
inpainting Loss $F1$	80.99	80.99	56.33	46.94	51.20	47.18	3.62
inpainting Loss MCC	81.63	81.63	57.22	47.23	51.75	47.82	3.67
inpainting PPV_L $PPV_{L;0.5}$	86.06	86.06	61.40	46.63	53.00	49.68	4.80
inpainting PPV_L PPV_L	86.13	86.13	61.65	46.47	53.00	49.72	3.64
inpainting PPV_L $F1$	84.65	84.65	57.20	47.49	51.90	47.96	3.75
inpainting PPV_L MCC	85.36	85.36	59.18	46.95	52.36	48.71	3.30
inpainting $PPV_{L;0.5}$ $PPV_{L;0.5}$	86.06	86.06	61.40	46.63	53.00	49.68	3.57
inpainting $PPV_{L;0.5}$ PPV_L	86.13	86.13	61.65	46.47	53.00	49.72	3.64
inpainting $PPV_{L;0.5}$ $F1$	84.65	84.65	57.20	47.49	51.90	47.96	3.48
inpainting $PPV_{L;0.5}$ MCC	85.36	85.36	59.18	46.95	52.36	48.71	3.65
inpainting $F1$ $PPV_{L;0.5}$	76.24	76.24	50.28	53.80	51.98	47.20	3.45
inpainting $F1$ PPV_L	78.10	78.10	52.71	53.00	52.86	48.28	3.79
inpainting $F1$ $F1$	78.93	78.93	52.81	52.82	52.81	48.25	3.67
inpainting $F1$ MCC	78.93	78.93	52.81	52.82	52.81	48.25	3.74
inpainting MCC $PPV_{L;0.5}$	76.24	76.24	50.28	53.80	51.98	47.20	2.81
inpainting MCC PPV_L	78.10	78.10	52.71	53.00	52.86	48.28	3.79
inpainting MCC $F1$	78.93	78.93	52.81	52.82	52.81	48.25	3.43
inpainting MCC MCC	78.93	78.93	52.81	52.82	52.81	48.25	3.73
jigsaw Loss $PPV_{L;0.5}$	66.80	66.80	46.06	35.42	40.05	35.42	4.61
jigsaw Loss PPV_L	66.41	66.41	46.07	35.41	40.04	35.42	4.94
jigsaw Loss $F1$	65.13	65.13	44.75	35.72	39.73	34.86	2.74
jigsaw Loss MCC	65.13	65.13	44.75	35.72	39.73	34.86	2.65
jigsaw PPV_L $PPV_{L;0.5}$	68.46	68.46	46.20	41.47	43.71	38.65	4.56
jigsaw PPV_L PPV_L	69.11	69.11	45.74	41.57	43.55	38.43	4.91
jigsaw PPV_L $F1$	68.91	68.91	45.90	41.58	43.63	38.53	2.78
jigsaw PPV_L MCC	68.91	68.91	45.90	41.58	43.63	38.53	3.64
jigsaw $PPV_{L;0.5}$ $PPV_{L;0.5}$	68.46	68.46	46.20	41.47	43.71	38.65	5.44
jigsaw $PPV_{L;0.5}$ PPV_L	69.11	69.11	45.74	41.57	43.55	38.43	4.50
jigsaw $PPV_{L;0.5}$ $F1$	68.91	68.91	45.90	41.58	43.63	38.53	2.46
jigsaw $PPV_{L;0.5}$ MCC	68.91	68.91	45.90	41.58	43.63	38.53	2.75
jigsaw $F1$ $PPV_{L;0.5}$	63.33	63.33	41.27	41.55	41.41	35.72	4.13
jigsaw $F1$ PPV_L	62.75	62.75	40.91	41.73	41.32	35.58	4.60
jigsaw $F1$ $F1$	62.68	62.68	40.46	41.78	41.11	35.32	2.89
jigsaw $F1$ MCC	62.68	62.68	40.46	41.78	41.11	35.32	2.79
jigsaw MCC $PPV_{L;0.5}$	63.33	63.33	41.27	41.55	41.41	35.72	4.07
jigsaw MCC PPV_L	62.75	62.75	40.91	41.73	41.32	35.58	4.71
jigsaw MCC $F1$	62.68	62.68	40.46	41.78	41.11	35.32	3.62
jigsaw MCC MCC	62.68	62.68	40.46	41.78	41.11	35.32	3.82
contrastive Loss $PPV_{L;0.5}$	74.18	74.18	47.47	50.58	48.97	43.89	3.77
contrastive Loss PPV_L	74.18	74.18	47.47	50.58	48.97	43.89	3.65
contrastive Loss $F1$	74.25	74.25	47.55	50.72	49.09	44.01	2.86
contrastive Loss MCC	74.25	74.25	47.55	50.72	49.09	44.01	3.73
contrastive PPV_L $PPV_{L;0.5}$	84.14	84.14	58.19	44.74	50.59	46.96	2.69

Task + checkpoint metric(s)	$\mathrm{PPV}_{L;0.5}$ / %	PPV_L / %	PPV / %	SEN / %	F1 / %	MCC / %	Energy / Wh
contrastive PPV_L PPV_L	84.71	84.71	59.83	43.91	50.65	47.32	3.62
contrastive PPV_L $F1$	81.95	81.95	49.32	46.61	47.93	43.07	3.43
contrastive PPV_L MCC	84.14	84.14	58.19	44.74	50.59	46.96	3.67
contrastive $PPV_{L;0.5}$ $PPV_{L;0.5}$	84.14	84.14	58.19	44.74	50.59	46.96	3.63
contrastive $PPV_{L;0.5}$ PPV_L	84.71	84.71	59.83	43.91	50.65	47.32	3.85
contrastive $PPV_{L;0.5}$ $F1$	81.95	81.95	49.32	46.61	47.93	43.07	3.68
contrastive $PPV_{L;0.5}$ MCC	84.14	84.14	58.19	44.74	50.59	46.96	3.04
contrastive $F1$ $PPV_{L;0.5}$	72.64	72.64	47.54	51.50	49.44	44.38	2.94
contrastive $F1$ PPV_L	72.64	72.64	48.19	51.38	49.73	44.73	3.53
contrastive $F1$ $F1$	73.15	73.15	48.05	51.38	49.66	44.64	3.75
contrastive $F1$ MCC	73.15	73.15	48.05	51.38	49.66	44.64	2.86
contrastive MCC $PPV_{L;0.5}$	75.59	75.59	47.49	52.51	49.88	44.83	2.97
contrastive MCC PPV_L	76.36	76.36	47.71	52.51	50.00	44.97	3.52
contrastive MCC $F1$	75.72	75.72	47.45	52.49	49.85	44.79	4.54
contrastive MCC MCC	75.72	75.72	47.45	52.49	49.85	44.79	2.61
bootstrap Loss $PPV_{L;0.5}$	80.80	80.80	52.03	51.72	51.87	47.23	3.70
bootstrap Loss PPV_L	80.35	80.35	50.85	51.95	51.39	46.64	3.13
bootstrap Loss $F1$	79.58	79.58	50.38	52.18	51.27	46.47	3.10
bootstrap Loss MCC	79.58	79.58	50.38	52.18	51.27	46.47	2.85
bootstrap PPV_L $PPV_{L;0.5}$	84.01	84.01	59.26	43.42	50.12	46.75	3.44
bootstrap PPV_L PPV_L	84.33	84.33	61.76	42.88	50.61	47.68	3.19
bootstrap PPV_L $F1$	83.69	83.69	58.69	43.63	50.05	46.58	2.71
bootstrap PPV_L MCC	84.01	84.01	59.26	43.42	50.12	46.75	3.85
bootstrap $PPV_{L;0.5}$ $PPV_{L;0.5}$	84.01	84.01	59.26	43.42	50.12	46.75	3.71
bootstrap $PPV_{L;0.5}$ PPV_L	84.33	84.33	61.76	42.88	50.61	47.68	4.64
bootstrap $PPV_{L;0.5}$ $F1$	83.69	83.69	58.69	43.63	50.05	46.58	2.69
bootstrap $PPV_{L;0.5}$ MCC	84.01	84.01	59.26	43.42	50.12	46.75	3.65
bootstrap $F1$ $PPV_{L;0.5}$	74.69	74.69	49.47	48.51	48.99	44.11	3.72
bootstrap $F1$ PPV_L	74.31	74.31	48.66	48.50	48.58	43.61	3.76
bootstrap $F1$ $F1$	74.69	74.69	49.47	48.51	48.99	44.11	3.66
bootstrap $F1$ MCC	74.69	74.69	49.47	48.51	48.99	44.11	2.68
bootstrap MCC $PPV_{L;0.5}$	74.69	74.69	49.47	48.51	48.99	44.11	3.73
bootstrap MCC PPV_L	74.31	74.31	48.66	48.50	48.58	43.61	2.92
bootstrap MCC $F1$	74.69	74.69	49.47	48.51	48.99	44.11	2.74
bootstrap MCC MCC	74.69	74.69	49.47	48.51	48.99	44.11	3.32

SUPPLEMENTARY NOTE VIII. ADDITIONAL EXAMPLE

Figures 5 and 6 show an additional example to the one in the main text. The cluster of false positive predictions seems to originate from the presence of another molecule in the reference of 5di2. If that molecule is not present the gap might be closed as predicted by our model. To deal with this situation consistently, either the dataset should include strictly monomeric structures, or multimeric structures would have to explicitely included and represented sufficiently in the dataset so the model has an opportunity to learn these intricacies.

---

[1] I. Kalvari, E. P. Nawrocki, J. Argasinska, N. Quinones-Olvera, R. D. Finn, A. Bateman, and A. I. Petrov, Non-coding RNA analysis using the Rfam database, Current protocols in bioinformatics **62**, e51 (2018).

[2] I. Kalvari, E. P. Nawrocki, N. Ontiveros-Palacios, J. Argasinska, K. Lamkiewicz, M. Marz, S. Griffiths-Jones,

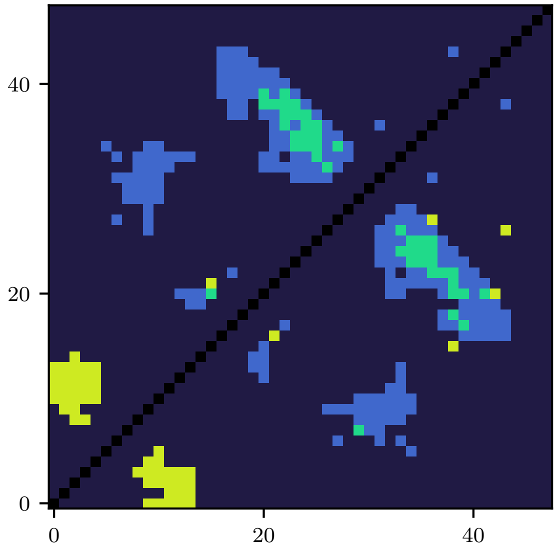

SUPPLEMENTARY FIGURE 5. Contact map (PDB: 5di2) - unfrozen vs. frozen. The upper left part shows the top L contact predictions for the best model with unfrozen backbone, the lower right one for the best model with frozen backbone. Green pixels refer to true positives, yellow to false positives, light blue to false negatives, and dark blue to true negatives.

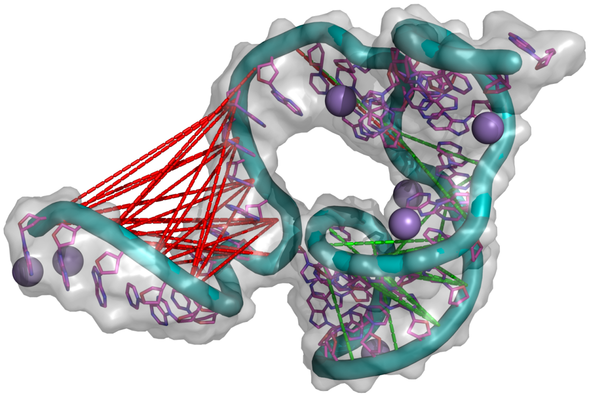

SUPPLEMENTARY FIGURE 6. 3D visualization of an RNA (PDB: 5di2). Green dashed lines indicate correctly predicted inter-residue contacts, red ones refer to false positives.

C. Toffano-Nioche, D. Gautheret, Z. Weinberg, *et al.*, Rfam 14: expanded coverage of metagenomic, viral and microRNA families, Nucleic Acids Research **49**, D192 (2021).

[3] Z. Weinberg, C. E. Lünse, K. A. Corbino, T. D. Ames, J. W. Nelson, A. Roth, K. R. Perkins, M. E. Sherlock, and R. R. Breaker, Detection of 224 candidate structured RNAs by comparative analysis of specific subsets of intergenic regions, Nucleic acids research **45**, 10811 (2017).

[4] M. B. Zerihun, F. Pucci, and A. Schug, CoCoNet—boosting RNA contact prediction by convolutional neural networks, Nucleic acids research **49**, 12661 (2021).

[5] F. Pucci, M. B. Zerihun, E. K. Peter, and A. Schug, Evaluating DCA-based method performances for RNA contact prediction by a well-curated data set, RNA **26**, 794 (2020).

[6] E. P. Nawrocki and S. R. Eddy, Infernal 1.1: 100-fold faster RNA homology searches, Bioinformatics **29**, 2933 (2013).

[7] T. Chen, S. Kornblith, M. Norouzi, and G. Hinton, A simple framework for contrastive learning of visual representations, in *Proceedings of the 37th International Conference on Machine Learning* (PMLR, 2020) pp. 1597–1607.

[8] T.-Y. Lin, P. Goyal, R. Girshick, K. He, and P. Dollár, Focal loss for dense object detection, in *2017 IEEE International Conference on Computer Vision (ICCV)* (2017) pp. 2999–3007.

[9] C. H. Sudre, W. Li, T. Vercauteren, S. Ourselin, and M. Jorge Cardoso, Generalised dice overlap as a deep learning loss function for highly unbalanced segmentations, in *Deep Learning in Medical Image Analysis and Multimodal Learning for Clinical Decision Support* (Springer International Publishing, 2017) pp. 240–248.

[10] R. K. Vinayak and R. Gilad-Bachrach, DART: Dropouts meet Multiple Additive Regression Trees, in *Proceedings of the Eighteenth International Conference on Artificial Intelligence and Statistics* (PMLR, 2015) pp. 489–497.

[11] R. M. Rao, J. Liu, R. Verkuil, J. Meier, J. Canny, P. Abbeel, T. Sercu, and A. Rives, MSA Transformer, in *Proceedings of the 38th International Conference on Machine Learning*, Proceedings of Machine Learning Research, Vol. 139 (PMLR, 2021) pp. 8844–8856.

[12] Will be provided upon publication.

[13] Y. Yang, X. Li, H. Zhao, J. Zhan, J. Wang, and Y. Zhou, Genome-scale characterization of rna tertiary structures and their functional impact by rna solvent accessibility prediction, Rna **23**, 14 (2017).

[14] M. Larkin, G. Blackshields, N. Brown, R. Chenna, P. McGettigan, H. McWilliam, F. Valentin, I. Wallace, A. Wilm, R. Lopez, J. Thompson, T. Gibson, and D. Higgins, Clustal W and Clustal X version 2.0, Bioinformatics **23**, 2947 (2007), https://academic.oup.com/bioinformatics/article-pdf/23/21/2947/49823064/bioinformatics_23_21_2947.pdf.
