# Supplementary material for: RNA contact prediction by data efficient deep learning
Source: Commun Biol. 2023 Sep 6;6:913. doi: 10.1038/s42003-023-05244-9 (PMC10482910; doi:10.1038/s42003-023-05244-9)
Supplement: Supplementary file 2 — Description of Additional Supplementary Files [file 42003_2023_5244_MOESM2_ESM.docx]

**Description of Additional Supplementary Files**

**File name:** Supplementary Notes

**Description:** Expanded method and additional results

**File name:** Supplementary Data Figure 2

**Description:** Raw data for figure 2

**File name:** Supplementary Data Figure 3

**Description:** Raw data for figure 3
